# Developing the clinical components of a complex intervention for a glaucoma screening trial: a mixed methods study

**DOI:** 10.1186/1471-2288-11-54

**Published:** 2011-04-21

**Affiliations:** 1 Health Services Research Unit, University of Aberdeen, Aberdeen, AB25 2ZD, UK

## Abstract

**Background:**

Glaucoma is a leading cause of avoidable blindness worldwide. Open angle glaucoma is the most common type of glaucoma. No randomised controlled trials have been conducted evaluating the effectiveness of glaucoma screening for reducing sight loss. It is unclear what the most appropriate intervention to be evaluated in any glaucoma screening trial would be. The purpose of this study was to develop the clinical components of an intervention for evaluation in a glaucoma (open angle) screening trial that would be feasible and acceptable in a UK eye-care service.

**Methods:**

A mixed-methods study, based on the Medical Research Council (MRC) framework for complex interventions, integrating qualitative (semi-structured interviews with 46 UK eye-care providers, policy makers and health service commissioners), and quantitative (economic modelling) methods. Interview data were synthesised and used to revise the screening interventions compared within an existing economic model.

**Results:**

The qualitative data indicated broad based support for a glaucoma screening trial to take place in primary care, using ophthalmic trained technical assistants supported by optometry input. The precise location should be tailored to local circumstances. There was variability in opinion around the choice of screening test and target population. Integrating the interview findings with cost-effectiveness criteria reduced 189 potential components to a two test intervention including either optic nerve photography or screening mode perimetry (a measure of visual field sensitivity) with or without tonometry (a measure of intraocular pressure). It would be more cost-effective, and thus acceptable in a policy context, to target screening for open angle glaucoma to those at highest risk but for both practicality and equity arguments the optimal strategy was screening a general population cohort beginning at age forty.

**Conclusions:**

Interventions for screening for open angle glaucoma that would be feasible from a service delivery perspective were identified. Integration within an economic modelling framework explicitly highlighted the trade-off between cost-effectiveness, feasibility and equity. This study exemplifies the MRC recommendation to integrate qualitative and quantitative methods in developing complex interventions. The next step in the development pathway should encompass the views of service users.

## Background

Complex interventions are widely used in the health service context but are well known to be difficult to develop and evaluate [1]. In the development phase the likely mechanism of action for the intervention should be understood with consideration given to implementation at an early stage (by consulting with key stakeholders) and to model the potential costs and effects of the proposed intervention. These factors can then be used to inform decisions such as whether a full scale trial would be appropriate or not (e.g. trial would have to be unfeasibly large; interventions are not likely to be cost-effective). The guidance by Craig and colleagues[1] highlights the potential of mixed methodologies to achieve these aims, in particular the role of qualitative enquiry to articulate the intervention's complexity, and the use of quantitative modelling to identify configurations of complex services that could potentially be cost-effective early in the development pathway. However, there are limited reports of how this augmented approach has been applied in practice. This paper presents the first phase of the development of a complex intervention for a potential national glaucoma screening trial for open angle glaucoma.

Glaucoma is a leading cause of avoidable blindness worldwide [2], and second to macular degeneration is the most common cause of blindness in the UK [3]. Open angle glaucoma is the most common form of glaucoma [2] and early detection and treatment is effective at reducing progressive disease [4,5]. However, the current UK practice of opportunistic case finding delays identification of the majority of cases [6] and late detection is a major risk factor for glaucoma blindness [7,8]. The two main reasons for under detection of glaucoma are poor uptake of community eye care services by members of the public [9-11] or missed cases by primary eye care services[12-15]. Based on the public health importance of glaucoma, it has been considered as a condition that might merit a population based screening programme. However, prior to initiating a screening programme several criteria need to be met concerning the condition, the test, the treatment and the screening programme. Importantly any benefits of screening must outweigh potential harms [16].

The UK National Screening committee (UKNSC), the body that advises the NHS on screening policy, considered glaucoma against these criteria in 2006 and concluded that there was insufficient evidence to recommend glaucoma screening, but that further research was required [17]. A health technology assessment (HTA) review was commissioned to inform a subsequent policy review on glaucoma screening. The HTA report [6] identified that no randomised controlled trials (RCTs) had been conducted to evaluate the effectiveness of glaucoma screening for reducing sight loss [18]. The economic modelling showed that any general population screening programme, based on age selection alone, was unlikely to be cost-effective (given current decision-making criteria in the UK [19]), but that 'targeted' screening of high risk groups may be. However, due to the limited primary data available, further research was advocated to test the model estimates and inform decisions about best practice for screening [20]. A separate HTA report, conducted in Finland, concluded that an organised screening programme for glaucoma could be a cost-effective strategy, and, in contrast to the report by Burr and colleagues [6,20] that screening was more likely to be cost-effective in older age groups [21]. The differences in conclusions were driven by differences in model structure and costs. Specifically, in the Finnish model the assumed gain in quality of life due to screening preventing inappropriate treatment (and hence side effects of treatment) in the elderly was a main driver for the cost-effectiveness of screening.

Prompted by the continuing uncertainty around whether to progress with a screening programme (and by the acknowledged public health importance of glaucoma), the international ophthalmic community has called for a high quality study evaluating population screening for glaucoma versus the current practice of opportunistic case finding [22-25]. Designing a high quality RCT of a screening intervention for open angle glaucoma presents particular challenges including: which screening test to use and who should administer it; where to locate the screening; which population to target; how to identify individuals in the target population; how to maximise attendance for screening; and how potential harms and benefits of screening in the glaucoma context should be considered.

The aim of this study was to inform the development of a glaucoma screening intervention that would be feasible and acceptable both in terms of service delivery and cost-effectiveness in a UK health service context. We explored opinions representing a service provider and policy perspective, namely eye-care providers, policy makers and health service commissioners, on the most feasible and acceptable components (target population, site, screening tests and operator) of glaucoma screening strategies. These insights provide key information to refine and revise an economic model initially developed by Burr and colleagues[6,20].

## Methods

We used an integrated qualitative and quantitative methods design. There are many screening tests and combinations of tests, test operators and screening locations that could be used in a glaucoma screening programme. Possible screening tests were identified and evaluated in a previous systematic review of screening test performance [26]. No single test or combination of tests was clearly superior. Selection of an optimal test or test combination therefore rests on the feasibility and acceptability to both users and service providers. A Delphi survey, developed from the previous HTA review and the review of screening test performance [6,20], with a sample of glaucoma experts worldwide, informed a reduced set of potential screening tests and testing arrangements. The findings from the Delphi study are being written up elsewhere; in brief, there was some agreement on the age of fifty to initiate screening in a primary care setting with ophthalmic trained technical assistants (hereafter referred as 'technicians') delivering the screening tests; however a wide range of screening tests and combinations of tests were selected with no agreement on what would be a suitable screening test.

To determine the feasibility and acceptability of a range of possible tests and testing arrangements in a UK National Health Service context we:

a) undertook semi-structured interviews with eye-care providers, policy makers and health service commissioners to elicit their experiences and perspectives about the main properties of a potential intervention for a national glaucoma screening trial; and

b) assessed the likely cost-effectiveness of proposed test strategies.

Detailed methods are presented below.

### Elicitation of provider perspectives on a potential screening intervention

#### Recruitment and sampling

The purposive sample for this qualitative component was selected to represent a wide range of health professionals including ophthalmologists, optometrists, general practitioners (GPs), nurses, technicians and directors of public health from across the UK (target numbers, together with numbers interviewed, are presented in Table 1). We liaised with Vision 2020 UK (an umbrella organisation focusing on visual impairment and aiming to facilitate greater collaboration and co-operation between organisations within the UK [27]), the relevant specialist royal colleges, the UKNSC, and the National Diabetic Retinopathy Screening Programme to facilitate purposive recruitment of a wide range of expert groups from the different organisational sites across the UK. Where possible, we included individuals working in urban and rural areas to reflect a broad range of provision. The leads of these organisations contacted members by email, providing a brief summary of the project and inviting them for interview. From this we received a list of potential participants as follows: 28 ophthalmologists, 31 optometrists, 6 GPs, and 12 nurses, whom we contacted by telephone and email to discuss the study in more detail, obtain informed consent and to arrange a suitable interview time. As is common in recruitment practice, we also received a small number of participants through the snowballing method - in particular leading to contacts in the 'technician' group (ophthalmic photographers and perimetrists) and with policy makers. In total we recruited a purposive sample of 46 (Table 1). Respondents were given the choice of telephone interviews or face-to-face interviews (to minimise bias on the basis of geographical accessibility).

**Table 1:** Interview respondent characteristics by profession, geographic and organisational designation

Provider Group	No. sought	No. interviewed	Location/organisational designation of interviewees
Ophthalmologists	10	13	**England**: working in Hospital Eye Service (HES) in general hospital (8) working in specialist eye hospitals (3)
			**Scotland**: working in HES in general hospital (2)

Optometrists	10	12	**England**: working in HES in general hospital (3)working in high street (2) working in both HES (general hospital) and high street (1) working in high street and as academic optometrist (1)
			**Scotland**: working in high street (2) working in high street and in HES in a general hosp (1)
			**Wales**: working in high street (1)
			**Northern Ireland**: working in high street (1)

Nurses	10	5	**England**: working in HES (1) working in specialist hospital (1)
			**Scotland**: working in HES (1) working in Diabetic Retinopathy Service (1) + a nurse with a shared remit for **England and Scotland**: working in voluntary sector

General Practitioners	10	4	**England**: practicing GP with a special interest in ophthalmology (1)
			**Scotland**: practicing GP also working as a clinical assistant in ophthalmology (HES) (1) practicing GP and academic GP (1) practicing GP with special interest in ophthalmology (1)

Technicians	5	3	To protect respondents' anonymity geographical location is not indicated here. Two medical photographers and one tester/grader

Policy, commissioning, planning and strategy	12	9	**England**: planning, policy development (4)
			**Scotland**: public health consultant, commissioners of public health services, clinical services (3)
			**England and Scotland**: joint remit for eye health prevention and policy working in voluntary sector (2)

#### Interviews

Interviews were conducted by three qualitative researchers (AG, DH, SM) and took place between January-October 2009. The focus of the interviews was on interviewees' perspectives about the main properties of their preferred screening trial intervention, specifying key criteria for feasibility and acceptability in a service context. In particular, we explored the reasons for the varying opinions around the choice of a screening test, and the broader contextual background to interviewees' opinions, as a way of identifying external factors that might be anticipated to impact on implementation. The purpose of this was to optimise the design of the intervention.

#### Data handling and analysis

Interviews were recorded, transcribed verbatim and anonymised. Field notes were taken during the interviews and immediately afterwards. We undertook a thematic qualitative analysis in accordance with constant comparative techniques derived from grounded theory [28]. We coded transcripts independently and jointly to identify common themes. Data analysis was a continuous iterative process with categorisation refined to reflect new themes and variations on existing themes. A thematic framework was developed consisting of six higher order themes, and two subsidiary branches.

To present the data in its most accessible form and to assist its utilisation in the synthesis with the subsequent phase of the project, we presented the data in two parts. The first part presented a systematic summary of opinions about preferred components of the intervention the screening properties - nominated tests, locations, test operators and diagnostic pathways (illustrated in Table 2). The second part presented an interpretative analysis of the broader implementation concerns - notably policy, institutional and organisational issues. Handling the substantial amount of data in this way facilitated the synthesis and integration of data into the economic model. Presenting the data in this way was, of necessity, a simplification of the full accounts; however, care was taken to ensure that the underlying relationships and links in the data were retained.

**Table 2:** Two examples of summaries of screening specifications representing diversity of provider views

Screen location	Screen operator	Target population	Screen tests	Criteria for screen positive	Diagnostic site for screen positives	Diagnostic assessor	Diagnostic test
Community (General Practice or optometry, or van in remote areas)	Optometrist or technician	60	Tonometry [GAT] + nerve fibre layer imaging [expressed preference for GDx as the nerve fibre layer imaging analysis technology] + perimetry [expressed a preference for Humphrey 24-2 and a strong dislike of FDT] ± anterior chamber depth [UBM].	Didn't say	Virtual clinic: Information from screen positives read by consultant ophthalmologist	Consultant ophthalmologist [strong dislike of this expert job being done by dedicated technicians, nurses, nurse consultants]	Reading of screen test information and then either discharged, retest or those reading positive referred for full standard care glaucoma work up and decision regarding treatment.

General Practice health centre	Nurse/technician or GP or self testing	50 but younger for African Caribbean ethnic groups	Ideally simple visual function test taking 2-3 minutes on laptop computer [Motion Detection Perimetry] and tonometry	Difficult to determine a cut -off threshold for IOP	HES	Expert	Full Glaucoma workup

HES: Hospital Eye Service IOP: Intraocular pressure GDx: Scanning laser perimetry

FDT: Frequency Doubling Technology UBM: Ultrasound biomicroscopy GAT: Goldmann Applanation Tonometry

### Economic modelling

One criterion used to judge whether an intervention should be taken forward to trial is whether it can potentially be cost-effective. The original economic model, [20], compared the cumulative costs and benefits (measured in clinical terms and quality adjusted life years) of two screening interventions with current practice. The model did not include a detailed specification of the screening tests but, due to the uncertainty regarding the optimal screening tests, incorporated the screening test performance of hypothetical tests. The summary data produced by the qualitative interviews provided new information to define further alternative screening strategies which in turn could be compared with current practice and tested in the economic model. For example, if an interviewee mentioned two potential alternative sites where screening could be conducted in a trial plus two alternative set of tests, four potential alternative screening strategies were thus deemed available for consideration within the economic model.

On the basis of the outputs from this model, the new screening strategies were then ordered according to the potential that they would be cost-effective. The ordered list of all possible trial screening strategies was reviewed by the multidisciplinary project management group (including ophthalmologists, statisticians, health economists, health psychologists and sociologists) with a view to identifying the most likely candidate strategy to take to trial. Likelihood was judged primarily on cost effective criteria but also considering feasibility criteria such that if screening were effective the interventions could be implemented into UK eye care services. In addition, the group also considered interventions that posed special circumstances e.g. interventions that:

a) could be developed from the non cost-effective alternatives that were not already considered in the table. For instance, if an interviewee mentioned a very high skilled operator together with a low cost set of tests while another stated a low skilled operator together with a very costly set of tests; these could be redeveloped into an alternative that looked more likely to be cost-effective (e.g. non-skilled operator together with a low cost set of tests).

b) were possibly cost-effective but were unrealistic (e.g. where a non skilled operator was mentioned together with a test for which a highly skilled operator was needed).

c) would be useful to consider in terms of their acceptability to patients and the public, and included tests in an early stage of development but likely to be candidate screening tests for evaluation in further research.

This study was classified as a service evaluation and did not require national research ethics committee approval (as advised by the North of Scotland Ethics Committee). We certify that all applicable institutional and governmental regulations concerning the ethical use of human volunteers were followed during this research.

## Results

### Interviews

The findings from the interviews identified many differing opinions on the choice of screening test (s), consistent with the prior Delphi study. The qualitative data provided insights into the difficulties agreeing on the screening test(s) and provided insights into how a screening intervention could be tailored according to particular groups who might not access eye care services. Of particular interest, and relevant to the potential implementation of a future screening programme, was concerns about professional tensions between expert provider groups in eye care services, and these concerns were reflected in their respective discussions about the feasibility and acceptability of the component parts (test, site, target population) of the intervention.

#### Preferred site

Most interviewees agreed that screening should take place in a primary care setting. Some thought that capitalising on current organisation and infrastructure in primary care, either in high street optometry or general practice, made most sense, although this was caveated by concerns about the business ethos of high street optometry, and how this may present a barrier to recruiting high risk groups:

'That's (private eye care services) probably going to gradually increase in a way because the likes of ... [specific company names], all the cheap and nasty services... they model and they tell their optometrists that they are expected to convert 80% of the people they see into a pair of glasses which means there's no wasting time with repeating examinations and doing the things that ... they may wish to do, you know time is money, you are here to flog glasses mate, get on with it, and that's very much ... the growing Business model'. (Ophthalmologist)

The reality of the commercial influences defining high street optometry featured in optometrists' responses. Currently there is variation among UK devolved administrations as to remuneration procedures for 'high street' (community) optometrists providing eye care services, with optometrists in Scotland currently receiving considerably more than optometrists in other areas of the UK:

'Currently there's no real incentive for optometrists to do it, so it's not a pre-requisite to get involved in glaucoma management or enhanced screening.' (Optometrist, England)

Some interviewees identified the positive appeal that placing general practitioners as key players in the programme represented. However, it was recognised that this was mitigated in practice by general practitioners' retreat from primary eye health, as government policy, and indeed the public, has increasingly identified optometry as the primary provider site of eye services in the community:

"...so on the one hand there's this move and I think a number of patients are getting used to it and accepting it, that when they've got an eye problem they go to the optician, they don't come to the GP. And it would kind of seem counterintuitive if then you've got this new system aimed at eye conditions which has gone back in GP surgeries, do you see what I mean?" (Practicing and academic GP)

The use of a mobile van was commonly endorsed as was the suggestion that the diabetic retinopathy screening service could be used as a possible template for glaucoma screening or indeed, that glaucoma screening could be piggybacked onto the existing screening for retinopathy.

#### Target population

A minority of interviewees highlighted the need for any screening programme to be directed towards the hard-to-reach groups, such as African and Caribbean ethnic groups. However, a larger number of the interviewees disagreed with this targeted approach - their own experiences as practitioners left them wondering whether it would be fairer to screen the whole population to ensure that no one with glaucoma was missed out. The dilemma that arose, therefore, was that what appeared to be the most cost-effective option [the targeted approach] was not necessarily the fairest choice, in terms of access to services. Below is an example of a respondent calling for a targeted approach to screening:

'We need to focus particularly our attentions on populations of people from black ethnic minority groups who have a higher susceptibility to certain eye disease and also to people on low incomes, those folk are furthest from engagement with the eye care sector' (policymaker)

Whereas another interviewee acknowledged the limited evidence to make judgements on whom to screen illustrating a difference in opinion between policy makers and providers:

'I think that's [universal screening at age 50] as good as anything because we've got very little data about what will happen if you go out to the community screening glaucoma. You don't know what's happening' (ophthalmologist)

#### Preferred Test(s) and preferred operator

Interviewees found it difficult to differentiate between identifying test(s) relevant for a *screening *intervention (ie a test to initially identify those more likely to have glaucoma) and those for the full *diagnosis *of glaucoma. Clinicians generally stressed the necessity of using three tests - visual field examination, measurement of intraocular pressure and imaging of the optic nerve - which reflects the practice of a diagnostic strategy used by many of the interviewees. A number also pointed out that the clinical grounds for recognition of glaucoma has undergone a revision, from one based on raised intraocular pressure to one that includes optic neuropathy:

''Optometrists... the training has improved and....also the dependence on intraocular pressure has been the main criteria. That has sort of shifted more towards visual fields and optic discs. I say it in that order because they still aren't very good at looking at optic discs. You know, but they do visual fields and they attempt to have a look at the optic disc..... and that has improved the detection rate. In time I think it's increased the number of false positives being referred to secondary care quite dramatically...."(Ophthalmologist)

Discussions about preferred tests highlighted differences in opinions between the two main clinical provider groups, ophthalmologists and optometrists. While clinician groups generally agreed that optometrists should have a key role in the delivery of tests, the majority indicated that the role of optometrists was to 'moderate' between the trained technical operators and the expert ophthalmic diagnostic role.

Overwhelmingly, and primarily out of financial consideration, clinicians suggested that 'technicians' supported by optometrists trained in glaucoma detection, represented the optimum choice of test operator. There were concerns raised, however, about ensuring adequate training for both technicians and optometrists - training together with quality assurance were identified as resource intensive essentials in any future screening trial. Unfortunately it was difficult to explore this in more detail as we had difficulties recruiting 'technicians' working in ophthalmic services to the study. Of the eight technicians we approached to take part only three were willing to be interviewed. However, we did have informal telephone discussions with a small number of technicians (who declined to formally participate in the study) who indicated their concerns regarding the lack of career structure for technicians, and the already intense workload that they experienced for little remuneration in often poor working conditions. This they saw as working against the premise that, in practice, there would be adequate technicians available, and committed to, resourcing a massive explosion of their workload should a screening programme be initiated. Their insights echoed those concerns expressed by the formally recruited technician group of respondents.

A number of optometrists (especially those who had combined primary and secondary care careers) raised concerns about the structural factors mitigating against high-street optometrists being able to access courses on glaucoma detection, notably that they received no appropriate reimbursement for the cost of the courses and time out of their commercial enterprises. Additionally, a number pointed to Scotland as the country in the UK where the interpretation of the optometrists' contract potentially provided more opportunity for specialising in glaucoma.

### Summary of the results of the interviews

The provider and policy perspectives regarding the configuration of an intervention for a national glaucoma screening trial indicated support for screening in primary care, using 'technicians' supported by optometrists. Potential barriers to implementation were identified. In particular the interview findings highlighted many of the complexities around a glaucoma screening programme such as the ethical issues of adopting a targeted approach to those in higher risk groups versus one of universal population provision, or on strategic and/or organisational grounds (priority setting in the NHS, adequate resourcing) and the associated constraints of a testing strategy. The study also flagged difficulties in being able to definitively identify preferred test(s), preferred operator, location and target population 'in principle and in isolation'. Rather, the choice of each of these configurations was seen to be dependent on a range of contextual factors. In particular, interviewees' opinions were influenced by their experiences of the current context of professional and organisational tensions in eye health services.

In summary:

• Preferred screening site was a primary care setting

• Preferred screening operator was a trained technician supported by a specialist optometrist

• Most interviewees thought that ultimately an ophthalmic consultant must make the diagnosis of glaucoma

• The preferred age to initiative screening was 50 years for the general population

• Most interviewees identified the 'at risk' groups as those with a primary family history, and those of African and Caribbean ethnic origin. Respondents indicated that the age at which screening were to be initiated should be under 50 for these risk groups.

• Potential intervention should have a low false positive rate

• Interviewees were aware of the finite resources of the NHS and the limitations this posed on service development, but nonetheless believed that a screening trial should occur because it could prevent people going blind

• The commercial culture of high street optometry was seen by many respondents as a barrier to capturing at risk groups. Current professional tensions between ophthalmologists and optometrists and changes in the policy and organisational determinants of eye care services that give optometrists increasing professional autonomy back, influenced respondents' preferred screening intervention properties and their broader discussions.

• Many interviewees, while advocating a screening trial, wanted the current infrastructure of case detection, referral refinement and shared care to be capitalised upon.

• Most ophthalmologists and optometrists in clinical practice were adamant that the screening tests must be based on current best clinical practice and that simplifying this protocol for a screening context was not acceptable practice

### Economic assessment of likely cost-effectiveness

All the potential screening components (i.e. nominated test(s), location(s), test operator(s), target population and diagnostic pathway(s)) put forward by the interviewees were summarised in tabular form for consideration in the economic model [20]. A total of 189 screening configurations were developed from interview data, further illustrating the difficulty in identifying the clinical components of a screening intervention. The multidisciplinary project management group (PMG) agreed there were no additional relevant options that could be developed from combination of components mentioned by different interviewees (consideration a. - see methods section). From this comprehensive list, a subset was immediately rejected as candidates for a screening intervention on the basis of known (from the previous model) lack of cost-effectiveness; 57 because screening required three or more tests (complex testing) or equipment costing more than £25 per testing based on a cost-effectiveness threshold of £20-30,000 per Quality Adjusted Life Year gained and 40 on the basis that the screening be undertaken by a highly trained health professional. The original economic modelling evaluation suggested that screening for glaucoma was only likely to be cost-effective if the screening is targeted at forty year olds with additional risk factors rather than general population screening [20]; on this basis, a further 62 configurations which specified screening a general population would have been ruled out. However, it was apparent from the qualitative interviews that an equity argument was voiced in favour of general population screening and considerations should be given to screening in remote rural locations. Both cost-effectiveness and equity arguments are likely to be important to decision-makers and taking into account the findings from the economic model and the interviews the PMG agreed that the 30 remaining configurations were realistic (consideration b. - see methods section). Seven of the remaining configurations considered a mobile unit as the setting where screening would be conducted. We considered this a variation of a primary care setting particularly relevant for remote areas. Key features of the remaining 23 configurations were variations on screening location including 'high street' optometry or in general medical practice. The tests were optic nerve photography or perimetry (a measure of visual field sensitivity) with or without tonometry (a device to measure intraocular pressure), operated by paramedical staff (e.g. nurse, technician or self-assessment, the latter being possible for perimetry available on Personal Computers and self assessment tonometers.

Integrating the findings from the qualitative interviews with the modelling thus identified the components of the screening test intervention that could be implemented in a trial from a service perspective. These are general population screening, for a cohort at age forty (based on findings of the qualitative interviews of the need to balance feasibility and equity with cost-effectiveness criteria) in a primary health care setting. Screening would be conducted by ophthalmic trained technical assistants, undertaking optic nerve photography or screening mode perimetry (a measure of visual field sensitivity) with or without tonometry. The precise location of screening a community setting would need to be tailored to local circumstances illustrated by the suggestion of using widely available eye care services e.g. the 'high street' optometrist or mobile units for remote areas.

## Discussion

This study identified screening interventions that eye care service providers, policy makers and health service commissioners would find feasible and acceptable to use in a trial of glaucoma screening and for implementation into practice if screening was effective. The economic modelling component of this study ruled out candidate tests that would be highly resource intensive and provided a short list of candidate strategies that had the greatest potential to be cost-effective. These involved testing in a primary care setting (because they are embedded in the community and have the infrastructure in place) delivered by technically trained personnel using a simple testing strategy - a measure of intraocular pressure (a known risk factor for glaucoma) [29], and either a measure of the visual field or photography of the optic nerve.

The screening configurations identified in this study meet one of the key principles of screening - that a simple, safe and affordable test is required before a screening programme can be implemented [30,16]. There is tension, however, when determining the most appropriate screening test, between a public health and a clinical perspective. The public health perspective requires that a screening test be sufficiently precise to distinguish those who probably do and do not have the disease at an acceptable cost, whereas the clinical view point often tends towards the desire for precision and reliability. There are many sophisticated imaging tests for diagnosing glaucoma, but these are not affordable for a public health intervention. Similarly, there are a variety of technologies to evaluate the peripheral visual fields. From the currently available tests, fundus photography or simple visual field testing combined with a measure of intraocular pressure meet the economic criteria. More precise technologies or examinations would then be required to examine those who screen positive. Currently, the recognition of glaucoma on fundus photography is a skilled task and would require manual grading by experienced observers. Automated grading systems for retinal images have been developed for diabetic retinopathy screening [31], and have been shown to be less costly and as effective as manual grading [32]. Similarly, there is potential for the development of automated systems for glaucoma recognition from retinal photographs [33]. The performance of photography, with and without automated grading, compared with simple visual field testing would need to be compared within the intervention arm in a future glaucoma screening trial or in a parallel companion study. Screening tests suitable for self-assessment are in the early stages of development, and should be considered as an option when the technologies have advanced sufficiently.

This study is one of the first to show the formal integration of qualitative and quantitative methodologies (as promoted in the MRC guidance) in the development of a complex intervention and specifically the use of a qualitative enquiry to restructure the care pathways of an economic model. The qualitative exploration enabled agreement to emerge for some aspects of the proposed intervention, whilst allowing reasons for disagreement to be explored in-depth. The detailed information that underpinned the lack of consensus (e.g. the disagreements between the professional groupings) informs the implementation planning for any of the proposed interventions, and aided interpretation of the results of the economic analysis in particular highlighting that a more universal coverage of screening all individuals above a certain minimum age would represent a more appropriate balance between equality of access and cost-effectiveness. The economic modelling facilitated the reduction of a wide selection of suggested interventions to a shortlist representing those most likely to be cost-effective. The study also brought together the perspectives of a wide range of stakeholders (including ophthalmologists, optometrists, GPs, nurses, technicians and policy makers) who would all have input into a screening programme for glaucoma, and this breadth of perspective adds to the veracity of our findings. Also, while previous attempts to provide high quality evidence for the case of screening for glaucoma have concentrated on the technical and technological aspects of design, evaluation and assessment, our research has also considered the implications of a screening programme on the complex organisational context in which the management of glaucoma exists.

Whilst our project was successful in facilitating the development of an intervention for future testing in a trial of glaucoma screening, it did have a number of limitations. In particular, recruitment proved more difficult in practice than had been hoped. Whilst ophthalmologists and optometrists were easier to recruit, nurses, technicians and general practitioners were more difficult. In the case of nurses and technicians, the difficulty appeared to be related to confidence in speaking as one who is located relatively low in the clinical 'hierarchy', whilst difficulties in recruiting general practitioners' in research is well recognised [34]. Additionally, despite the use of telephone interviews, we were unable to recruit as many respondents with practice experience of isolated and rural locations.

The MRC revised guidance outlines well the appropriate steps for the development of a complex intervention [1]. Integral to its tenets is the recommendation for the synthesis of qualitative and quantitative methods, as well as the need to consider implementation issues through participants' accounts of their perspectives and experiences. The integration process, however, required significant iteration between the disciplinary approaches to ensure that the qualitative data could be synthesised into an economic model. The specification of particular screening interventions was a challenge for the qualitative researchers because this was not a standard way of interpreting the data produced. Nevertheless, two complementary mechanisms to synthesising the qualitative data were used - firstly to systematically sort and describe the respondents' opinions about preferred components of the intervention (to allow direct usage in the economic model) and secondly to use the richness of the data provided by the qualitative data to undertake an interpretative analysis of the results of the economic model and around the implementation considerations in which the preferences were nested. The importance of interplay between disciplines in mixed methods research has been emphasised in the literature, particularly by O'Cathain and colleagues [35].

We have identified screening strategies that are acceptable in a service provider and policy context, but they may not be acceptable to health care users. Additional research is required to elicit the beliefs of health service users regarding the acceptability of these screening interventions and their current use of eye care services to identify the most likely behaviour change interventions for maximising attendance for glaucoma testing. These findings will provide additional information, in terms of potential uptake of any screening interventions and current uptake of current eye care services, to be integrated into the economic model to inform the feasibility of any future glaucoma screening trial. This research is underway.

## Conclusions

This paper has highlighted how mixed methods research can inform the development of an intervention for screening for open angle glaucoma. It allowed the identification of screening interventions that could be implemented in a UK health service context. Integration within an economic modelling framework explicitly highlighted the trade-off between cost-effectiveness, feasibility and equity. The next step in the intervention development is to seek the views of service users.

## Competing interests

The authors declare that they have no competing interests.

## Authors' information

**Members of the Glaucoma screening Platform Study group**:

***Writing committee for this paper: ***(in alphabetical order) Jennifer M Burr, Marion K Campbell, Susan E Campbell, Jillian J Francis, Alexandra Greene, Rodolfo Hernández, Debra Hopkins, Sharon K McCann and Luke D Vale. The writing committee have read and approved the final manuscript.

***Management group (additional to writing group members): ***(in alphabetical order) Augusto Azuara-Blanco, Maria Prior and Craig Ramsay

***Advisory Group: ***(in alphabetical order) Rustom Bativala, David Crabb, David Garway-Heath, Roger Hitchings; Stephen McPherson, Anja Tuulonen, Ananth Viswanathan, Heather Waterman and Richard Wormald.

## Authors' contributions

JMB, MKC, JJF, AG, RH, CR, and LV had the original ideas for the study. All authors developed the protocol. AG, DH, SMcC and conducted the interviews, DH, AG performed the qualitative analysis. RH performed the economic analysis. All authors contributed to critical revisions of the paper. AG is guarantor.

## Pre-publication history

The pre-publication history for this paper can be accessed here:

http://www.biomedcentral.com/1471-2288/11/54/prepub
